# Circulating TGF-β Pathway in Osteogenesis Imperfecta Pediatric Patients Subjected to MSCs-Based Cell Therapy

**DOI:** 10.3389/fcell.2022.830928

**Published:** 2022-02-09

**Authors:** Arantza Infante, Leire Cabodevilla, Blanca Gener, Clara I. Rodríguez

**Affiliations:** ^1^ Stem Cells and Cell Therapy Laboratory, Biocruces Bizkaia Health Research Institute, Cruces University Hospital, Barakaldo, Spain; ^2^ Service of Genetics, Cruces University Hospital, Barakaldo, Spain

**Keywords:** stem cells, cell therapy, TGF-β, osteogenesis imperfecta, mesenchymal stem cells

## Abstract

Osteogenesis Imperfecta (OI) is a rare genetic disease characterized by bone fragility, with a wide range in the severity of clinical manifestations. The majority of cases are due to mutations in *COL1A1* or *COL1A2*, which encode type I collagen. There is no cure for OI, and real concerns exist for current therapeutic approaches, mainly antiresorptive drugs, regarding their effectiveness and security. Safer and effective therapeutic approaches are demanded. Cell therapy with mesenchymal stem cells (MSCs), osteoprogenitors capable of secreting type I collagen, has been tested to treat pediatric OI with encouraging outcomes. Another therapeutic approach currently under clinical development focuses on the inhibition of TGF-β pathway, based on the excessive TGF-β signaling found in the skeleton of severe OI mice models, and the fact that TGF-β neutralizing antibody treatment rescued bone phenotypes in those OI murine models. An increased serum expression of TGF-β superfamily members has been described for a number of bone pathologies, but still it has not been addressed in OI patients. To delve into this unexplored question, in the present study we investigated serum TGF-β signalling pathway in two OI pediatric patients who participated in TERCELOI, a phase I clinical trial based on reiterative infusions of MSCs. We examined not only the expression and bioactivity of circulating TGF-β pathway in TERCELOI patients, but also the effects that MSCs therapy could elicit. Strikingly, basal serum from the most severe patient showed an enhanced expression of several TGF-β superfamily members and increased TGF-β bioactivity, which were modulated after MSCs therapy.

## Introduction

Osteogenesis Imperfecta (OI), a rare skeletal dysplasia with a high degree of genetic and phenotypic heterogeneity, is characterized by bone fragility, due to low bone mass and abnormalities in bone material properties. Accordingly, OI patients manifest an increased risk of fractures and skeletal deformities with a broad range of clinical severities: mild, moderate, severe and even lethal (Marini et al., 2017). OI is currently considered a collagen-related disorder, caused mainly by autosomal dominant mutations in type I collagen, the main bone extracellular matrix (ECM) protein (approximately 85% of all cases), or by mutations (autosomal dominant or recessive) in genes playing key roles in collagen homeostasis (around 15%) (Jovanovic et al., 2021). OI patients with mutations in *COL1A1* or *COL1A2* genes, who have a mixture of normal and abnormal collagen fibrils, can exhibit a wide range of OI severities, depending on the mutated α-chain and the type and position of the mutation along the triple helix (Marini et al., 2007). Thus, mutations that disrupt the structure of type I collagen (usually glycine substitutions) lead to severe OI, manifested in multiple low-trauma fractures throughout patient’s lifetime, short height, long-bone deformities, reduced mobility and chronic pain.

Currently, there is no effective treatment for OI. Since OI bones exhibit an increased bone remodeling, with a higher bone resorption at the expenses of bone formation, inhibitors of bone resorption, mainly bisphosphonates (BPs), are the first-line therapy in pediatric OI (Tauer et al., 2019). However, although BPs increase bone mineral density (BMD) in most OI patients, their efficacy in reducing long bones fractures and pain is controversial. Moreover, the associated adverse events (such as delayed bone healing of osteotomy site) and the safety about long-term use (BPs are retained in bone for extended periods after discontinuation of therapy) are a matter of concern. Hence, the development of new therapeutic strategies exploring novel safer and more effective approaches to address the pathological OI bone phenotypes is an actual and urgent need. In this line, the cell therapy based on MSCs emerged as a possible therapeutic option, with the assumption that MSCs would engraft in host bone and differentiate into osteoblasts, the collagen-producing cells, ameliorating the symptoms associated with OI (Pereira et al., 1995). Thus, MSCs therapy was first addressed by Horwitz and coworkers, who administered allogenic MSCs in immunosuppressed OI pediatric patients (Horwitz et al., 1999; Horwitz et al., 2002). One or two MSCs infusions were demonstrated to be feasible and safe, exerting clinical improvements of OI phenotypes, in spite of being short-lived with transitory beneficial effects, mainly because the expected cell engraftment into bone was utterly low (Horwitz et al., 1999; Horwitz et al., 2002; Götherström et al., 2014). The existence of a paracrine mediation of MSCs was then considered as underlying mechanism responsible for the observed clinical benefits in OI patients (Horwitz et al., 2002; Otsuru et al., 2018; Infante et al., 2021).

In order to overcome the transitory effect of MSCs therapy in OI pediatric patients, we conducted an independent, multi-center cell therapy phase I clinical trial based on reiterative infusions of allogenic MSCs applied to two OI pediatric patients (TERCELOI) (Infante et al., 2021). Moreover, to avoid a possible alloimmunization of non-immunosuppressed patients after repeated exposure to non-self MSCs, the need of human leukocyte antigen (HLA)-identical or histocompatible (5 shared out of six HLA antigens) not affected sibling donor was mandatory to enroll in TERCELOI. In fact, only two domestic patients fulfilled all the restricted inclusion criteria, P01 and P02 (Table 1). P01, a 6-year-old boy affected by severe Type III OI, carried a *de novo* heterozygous missense mutation in exon 16 of *COL1A1*, leading to a glycine substitution in the α1(I) chain of type I collagen. P02 (8-year-old girl affected by moderate Type IV OI), carried a *de novo* heterozygous variant in exon 35 of *COL1A2*, leading to the skipping of exon 35, in the *α*2(I) chain of type I collagen. We demonstrated that the reiterative cell therapy was safe and both patients showed durable improvements regarding the reduction of the number of bone fractures and enhancement in morphometric bone parameters, leading to a better quality of life. In TERCELOI, we also addressed for the first time, the possibility of a paracrine mechanism exerted by MSCs in OI patients’ context. Intriguingly, the clinical beneficial effects were especially noticeable in the most severe patient, P01, and after the first MSCs infusion, coupled with the molecular and cellular significances characterized by enhanced serum response in terms of global protein expression and pro-osteogenic capabilities among others (Infante et al., 2021). Moreover, P01, but not P02 basal serum also showed a clear upregulation of several bone-fracture associated miRNAs, which were gradually downregulated during the cell therapy.

**TABLE 1:** Characteristics of TERCELOI pediatric patients.

	Gender	Age (years)	OI type	Disease Severity	Affected Gene	Mutation	Molecular consequence
P01	Male	6	III	Severe	*COL1A1*	c.1031G > A	Glycine substitution p.Gly344Asp
P02	Female	8	IV	Moderate	*COL1A2*	c.2133+6T > A	Skipping of exon 35

Other completely different therapeutic strategy for OI, currently under clinical development, attempts to inhibit the transforming growth factor (TGF-β) signaling, a key pathway for bone homeostasis (MacFarlane et al., 2017). The *rationale* for this approach stands on the excessive TGF-β signaling found in the skeleton of three severe OI mouse models (*Col1a2* ^*+/G61*^ *°C*, *Crtap* ^*−/−*^ and *Col1a1* ^*Jrt/+*^), and the concomitant rescue of the pathological bone phenotypes by using 1D11, a specific anti-TGF-β monoclonal antibody in two of them, *Col1a2* ^*+/G61*^ *°C*, *Crtap* ^*−/−*^ (Grafe et al., 2014; Greene et al., 2021). Interestingly, the inhibition of TGF-β signaling by 1D11 was not effective in *Col1a1* ^*Jrt/+*^ mice (Tauer et al., 2018), suggesting that the different response to anti-TGF-β treatment depends on the severity of the OI mouse model, which in turn, is determined by the mutation type leading to OI. Thus, *Col1a1* ^*Jrt/+*^ mice carry a splice mutation in *Col1a1* leading to an 18-amino acid deletion in the collagen I alpha I chain, exhibiting the most severe phenotype within these three OI mice models (Tauer et al., 2018).

Surprisingly, the knowledge about the status of TGF-β pathway activation in OI patients is quite scarce, although some evidences, such as an increased expression of TGF-β receptors in human OI osteoblasts, point also to an increased TGF-β signaling in OI patients (Gebken et al., 2000). In this line, the pathogenic excessive TGF-β activation in OI could be correlated with increased circulating TGF-β levels, although to the best of our knowledge, this possibility has not been described in OI population. Supporting this assumption, correlations between increased circulating TGF-β levels and other pathologies with causative alterations in ECM components which lead to TGF-β dysregulation have been described, such as in Marfan syndrome, a genetic disease caused by mutations in *FBN1*, encoding fibrillin-1, an ECM protein that binds to latent TGF-β. Thus, mutations in fibrillin-1 leads to an increased pool of active TGF-β and therefore to an enhanced TGF-β signalling (Matt et al., 2009; Franken et al., 2013). Moreover, increased TGF-β circulating levels have also been observed in conditions altering bone homeostasis, such as extensive exercise and bone fractures healing (Hering et al., 2001; Hering et al., 2002; Zimmermann et al., 2005; Sarahrudi et al., 2011). In addition, evidences of a positive correlation between increasing circulating TGF-β levels and a decreased bone mineral density in osteoporosis, a low bone mass disease, have been also described (Grainger et al., 1999; Wu et al., 2013; Faraji et al., 2016).

**FIGURE 1:**
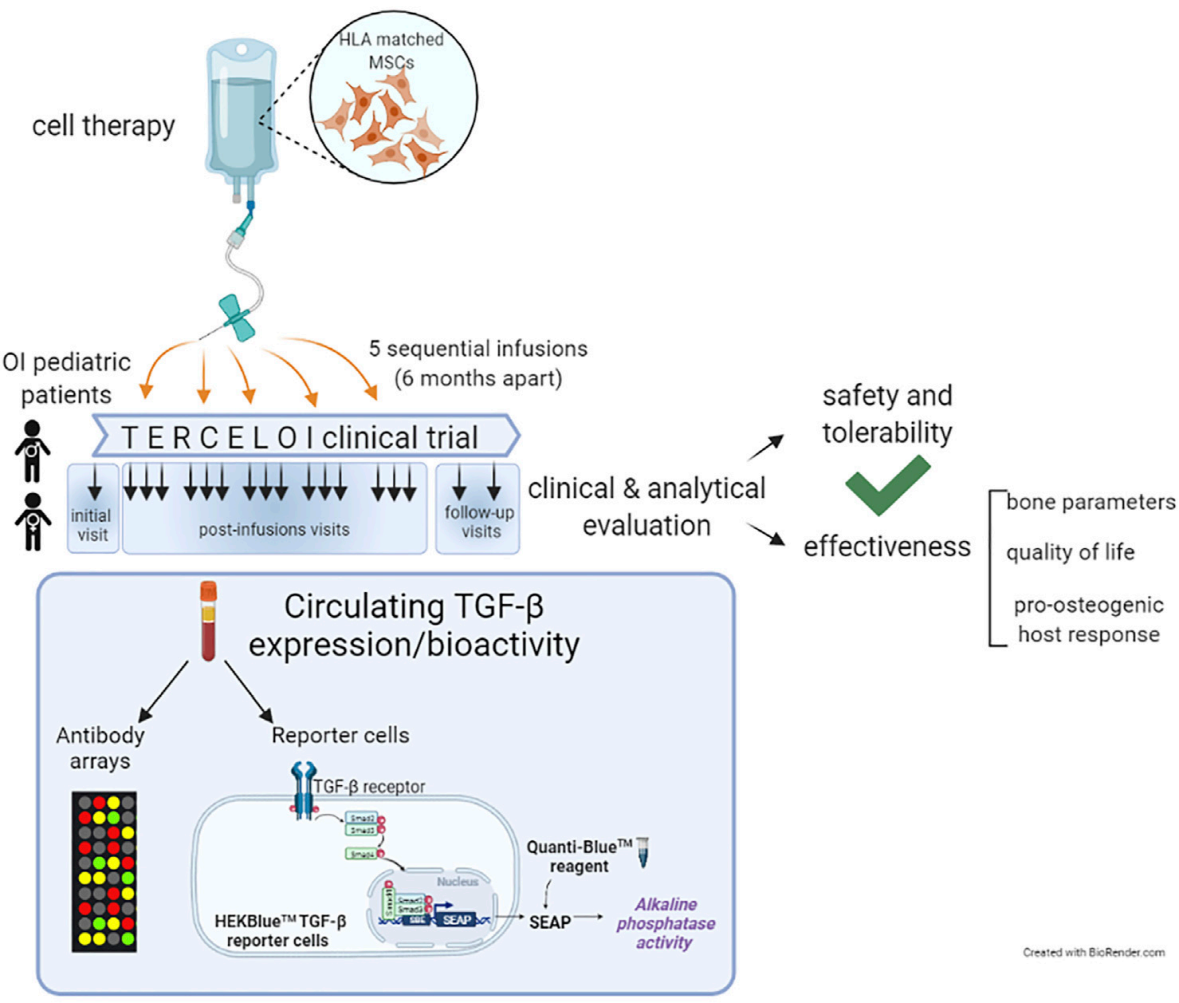
Schematic diagram illustrating TERCELOI clinical trial cellular infusions and the current study of circulating TGF-β expression and bioactivity in patients’ sera collected before (basal serum) and after the five consecutive MSCs infusions. The post-infusions sera included those collected 1 week (1w), 1 month (1 m) and 4 months (4 m) after each cell infusion, as well as the follow-up sera, collected 1 year and 2 years after the last cell infusion.

Considering these previous evidences, in this work we have delved into TERCELOI, exploring the circulating expression levels and functional activity (bioactivity), of TGF-β superfamily in OI patients before and after being subjected to the cell therapy (Infante et al., 2021). For this, we re-analyzed the sera proteomic data obtained in TERCELOI, based on antibody arrays which covered multiple members of the TGF-β superfamily such as structurally related ligands, TGF-β transmembrane receptors, and effectors. In order to elucidate the resultant bioactivity regarding TGF-β signaling, we also examined the ability of OI sera samples in activating TGF-β pathway, by using a TGF-β reporter cell line (Figure 1).

To the best of our knowledge, the current study is the first to address the circulating TGF-β pathway in the context of OI patients. Our results, which are indeed supported by the aforementioned molecular and cellular findings obtained from TERCELOI, show an increased expression and activation of TGF-β pathway in the basal serum from the most severe patient. Moreover, in the most severe patient, TGF-β signaling was modulated after MSCs therapy. These results suggest that different circulating TGF-β pathway activation could be occurring depending on OI mutation and its corresponding severity. Even more, MSCs therapy could modulate the enhanced TGF-β bioactivity in the case of the severe patient.

## Materials and Methods

### Ethics Statement

The study was in accordance with the ethical standards formulated in the Helsinki Declaration and was approved by the Basque Ethics Committee for Clinical Research and the Spanish Agency of Medicines and Medical Devices (AEMPS).

### Patients and Sera Samples

TERCELOI is a clinical trial Mesenchymal Stem Cell Therapy for the Treatment of Osteogenesis Imperfecta, registered at clinicaltrials.gov (NCT02172885) and eudract. ema.europa.eu (2012-002553-38). TERCELOI is an independent multi-center phase I clinical trial to evaluate the feasibility, safety and potential efficacy of infused sibling HLA-matched MSCs in non-immunosuppressed children with OI. Due to the highly restrictive eligibility criteria, especially limited by the need of having an HLA-matched unaffected sibling susceptible to donate, only two patients enrolled: P01, male, 6 years 1 month of age affected by severe OI and P02, female, 8 years and 1 month of age with a moderate OI. Patients’ characteristics are reported in Table 1.

Blood samples were collected in clot activator tubes (BD Vacutainer) and left undisturbed for 40 min at RT to allow the blood to clot. Then, blood samples were centrifuged at 1,300 g at RT during 15 min to remove the clot. The resulting serum supernatant was immediately aliquoted, frozen and stored at-80°C until analysis.

### Antibody Arrays

RayBio® biotin label-based (L-Series) Human Antibody Array 1,000 kit (AAH-BLG-1000), including a total of 1,000 soluble proteins, were used to perform sera hybridizations according to the manufacturer’s instructions (RayBiotech, United States) and as previously described(Infante et al., 2021). Sera from P01 and P02 collected before the cell therapy (basal serum) and 1 week, 1 month and 4 months after the first MSCs infusion were use. In order to be comparable, each array was processed under the same conditions and signals were scanned using an Axon GenePix laser scanner and data normalized with RayBiotech analysis tool. Fluorescent intensities were obtained by taking the mean of the two spots specific to each target protein, and all protein intensities on each array were normalized against the negative control, representing non-specific binding of the Cy3-conjugated streptavidin, and positive control spots, standardized amounts of biotinylated IgGs printed directly onto the array. Thus, after *subtracting background* signals spot intensities from each array, fluorescent signal intensities were normalized to *positive* controls, allowing the comparison among different arrays.

In this work, after normalization and to ensure the detection of a positive, real binding of target proteins to array antibodies, only the spots with a fluorescent intensity ≥300 above background were considered, as previously described by other studies using the same technology(Wang et al., 2020). To compare the TGF-β superfamily member expression after and before the cell therapy, any ≥1.5-fold increase or ≤0.65-fold decrease in signal intensity for a single protein between samples was considered a significant difference in expression.

### TGF-β Reporter Cell Line

The ability of patients’ sera to activate the TGF-β pathway was determined using the HEK-Blue-TGF-β^TM^ reporter cells (Invivogen) according to the manufacturer’s protocol. Briefly, 180 µL of HEK-Blue-TGF-β^TM^ reporter cells at a concentration of 280,000 cells/mL were seeded in p96 plates in test medium (DMEM 4.5 g/L glucose, 2 mM l-glutamine, 10% FBS heat inactivated, Pen-Strep 100 U/mL) in the presence of 20 µL of patient’s serum (10% v/v) for 24 h at 37°C and 5% CO_2_. Then, 20 µL of the cell culture supernantant were removed from each well and transferred to a transparent p96 plate containing 180 µL of prewarmed QUANTI-Blue solution and incubated at 37°C (in the dark) during 30 min. The QUANTI-Blue is a colorimetric enzymatic assay in which the presence of alkaline phosphatase changes the media color from pink to blue and can be quantified at 655 nm using a microplate reader. 20 µL of test medium and 20 µL of human recombinant TGF-β at 10 ng/ml were used as negative and positive controls, respectively. For each serum, the HEK-Blue-TGF-β^TM^ reporter cells were seeded in octuplicates. Two independent experiments were performed for each serum. Data are mean ± standard deviation obtained from the two independent experiments. Induced SEAP data for each serum is expressed normalized versus the values obtained for the negative control in each experiment.

## Results

### Increased Circulating TGF-β-Related Members Expression in Severe OI Patient

**FIGURE 2:**
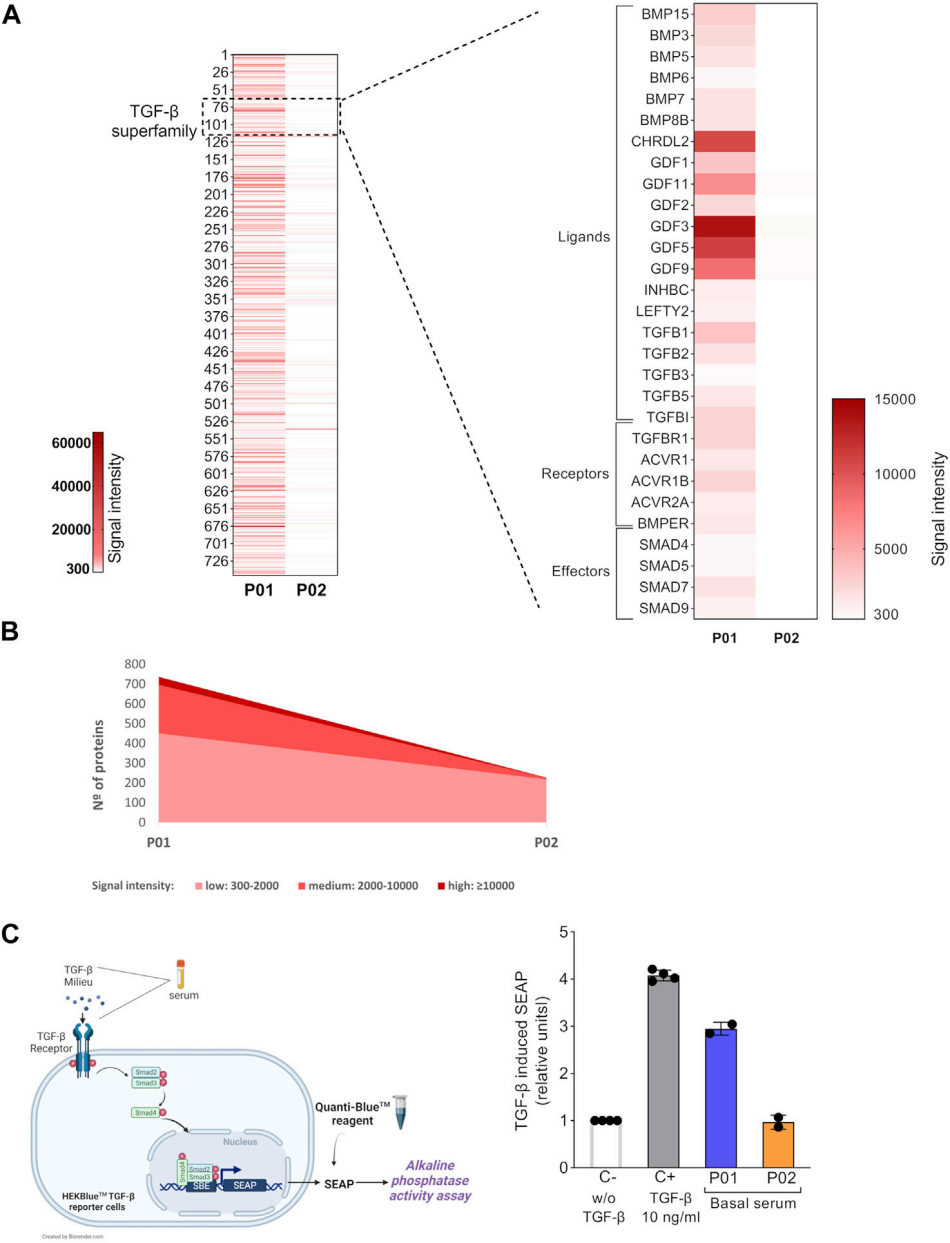
Basal circulating TGF-β superfamily members’ expression and bioactivity in OI patients. **(A)** Left heatmap shows the fluorescent signal intensities for target proteins expressed in P01 and P02 basal sera samples. Only the spots with a fluorescent intensity cutoff ≥300 above the background were considered. Right heatmap magnified shows the fluorescent intensities of the TGF-β superfamily members present in basal P01 and P02 sera are shown (right heatmap). **(B)** Graph showing the total number of expressed proteins in P01 and P02 basal sera, grouped according to their fluorescent signal intensity: low (300-2,000), medium (2,000-10,000) and high (>10,000). **(C)** Left, schematic representation of the HEK-Blue-TGF-β^TM^ reporter cell line assay. Right, Scatter plot with bars showing TGF-β-induced SEAP in HEK-Blue-TGF-β^TM^ cells. The TGF-β induced SEAP from patients’ sera samples is shown normalized *versus* that shown by the negative control (C-; w/o TGF-β). C+ stands for the positive control, cells stimulated with TGF-β (10 ng/ml). The experiments were performed in octuplicates for each condition and repeated two independent times. Data are mean ± standard deviation obtained from the two independent experiments. Each dot represents an independent experiment.

The study scheme is illustrated in Figure 1. First, we explored the global protein expression of basal OI serum (before the cell therapy), by using antibody array technology, which allows the simultaneous determination of over 1,000 proteins (Figures 2A,B). After detecting the expressed proteins in each basal serum (showing a fluorescent intensity of at least 300 above the background), we compared the total number of proteins expressed in the sera from P01 and P02 patients (Figure 2A, left heatmap). Interestingly, we observed a very different trend in terms of number and expression level between the two OI patients. Thus, 746 of the 1,000 proteins evaluated were detected as expressed in P01 (more than 70% of the total target proteins) *versus* the only 228 detected proteins in P02 (the ≈25% of total target proteins) (Figure 2A, left heatmap). Moreover, the fluorescent intensity of the detected proteins in P01 was in general higher than that exhibited by detected proteins in P02 basal serum, indicating a higher expression of circulating proteins in P01. Therefore, after grouping expressed proteins depending on fluorescent intensities, P01 basal serum showed 451 proteins with low signal intensities (signal between 300 and 2,000), 246 with medium signal intensity group (between 2,000 and 10,000) and 40 proteins with a high signal intensity (over 10,000). In the case of P02, the majority of the detected proteins, 218, exhibited a low signal intensity, whereas only eight and two proteins displayed medium and high signal intensities respectively (Figure 2B). Further analysis identified a quite high expression of a number of TGF-β-relevant members, including ligands (activators and inhibitors), receptors and effectors in P01 basal serum, contrarily to P02 basal serum (Figure 2A, right heatmap). These results point to the existence of a global higher expression of circulating TGF-β superfamily members in P01, finding that could be due to the highest OI severity exhibited by this patient.

### Increased TGF-β Bioactivity in Basal Serum From Severe OI Patient

The circulating TGF-β-enriched milieu in basal P01 serum, showed a coexistence of several TGF-β superfamily ligands (activators and inhibitors which can bind the same receptors and compete with each other for binding) and soluble receptors (able to bind to ligands and therefore to modulate the downstream TGF-β signaling) (Wang et al., 2017; Nickel et al., 2018; Martinez-Hackert et al., 2021). This mixture of activating and inhibiting signals led us to interrogate for the final TGF-β bioactivity of P01 (and P02) basal serum. For this, we used the HEK-Blue-TGF-β^TM^ reporter cell line which expresses TGFBRI, Smad3 and Smad4 genes and a secreted embryonic alkaline phosphatase (SEAP) reporter gene under the control of the *ß*-globin minimal promoter fused to three Smad3/4-binding elements (SBE). Stimulation of HEK-Blue-TGF-β^TM^ cells with TGF-β induces the activation of the TGF-β/Smad signaling pathway leading to the formation of a Smad3/Smad4 complex, which enters the nucleus and binds SBE sites inducing the production of SEAP. The secreted SEAP can be measured using QUANTI-Blue™ solution, a SEAP detection reagent (Figure 2C, left image). We first validated the functionality of this reporter system by stimulating the HEK-Blue-TGF-β^TM^ cells with the manufacturer’s recommended concentration of recombinant human TGF-β, which led to a high TGF-β pathway activation (Figure 2C, right). Concerning TERCELOI sera samples, we found that the TGF-β bioactivity of P01 basal serum was three times higher than that exhibited by P02, consistently with the TGF-β superfamily enrichment detected in P01 basal serum. In fact, P02 basal serum was unable to activate TGF-β pathway, showing similar activation levels to that obtained by the negative control (w/o TGF-β). Interestingly, P01 basal serum showed almost similar levels of TGF-β signaling activation as the positive control (Figure 2C, right). These data suggest that in the TGF-β enriched milieu of P01 basal serum, the TGF-β pro-activating factors prevailed over those inhibiting this pathway.

### The Circulating TGF-β Pathway in the Severe OI Patient Was Modulated After the MSCs Therapy

**FIGURE 3:**
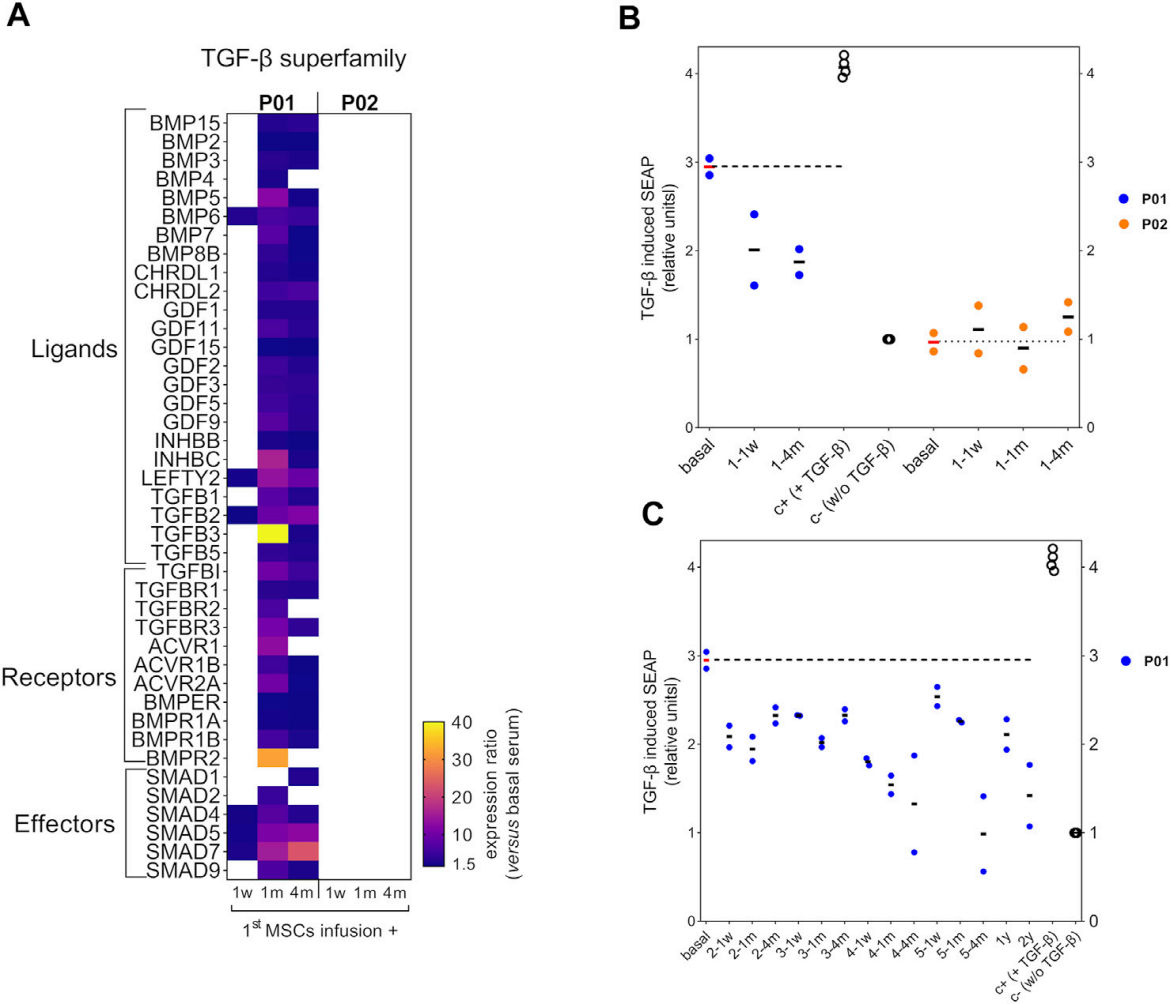
Circulating TGF-β superfamily members expression and bioactivity after MSCs therapy in OI patients. **(A)** Heatmap showing the expression ratio of TGF-β superfamily members after the 1st MSCs infusion in P01 and P02, calculated as the fluorescent signal intensity of target proteins after the MSCs therapy, 1 week (1w), 1 month (1 m) or 4 months (4 m) *versus* the fluorescent intensity of target proteins from each patient’s basal serum. To be considered, a cutoff ratio of ±1.5 was established. **(B)** Scatter plot showing TGF-β induced SEAP of HEK-Blue-TGF-β^TM^ cells exposed to P01 and P02 sera collected after the first MSCs infusion and compared to the results obtained with basal serum for each patient. The TGF-β induced SEAP is shown normalized *versus* the negative control. **(C)** Scatter plot showing TGF-β induced SEAP of HEK-Blue-TGF-β^TM^ cells exposed to the sera from P01 obtained during the 2nd, 3rd, 4th, 5th infusions and follow-up visits, and compared to the results obtained with basal serum. Two independent experiments were performed with P01 and P02 sera, each of one with their respective positive (c+) and negative controls (c-). Within each independent experiment, each condition was performed in octuplicates. Data are mean ± standard deviation obtained from the two independent experiments. Each dot represents an independent experiment.Dashed horizontal lines illustrate the TGF-β bioactivity level of basal P01 and P02 sera.

Next, we interrogated whether a modulation of the circulating TGF-β pathway could be occurring after the MSCs therapy in P01 and P02. Thus, we focused on the sera collected after the 1^st^ MSCs infusion, which, in the case of P01, showed in TERCELOI the most significant molecular findings in terms of global protein and miRNAs expression (Infante et al., 2021). We studied the antibody arrays’s signal intensities of TGF-β superfamily members in P01 and P02 sera collected 1 week, 1 month and 4 months after the 1st cell infusion, and compared them to those of their respective basal sera. Interestingly, we found in P01 a profound upregulation of the expression level of most TGF-β superfamily members analyzed, especially in the sera collected 1 and 4 months after the 1^st^ cell infusion (Figure 3A). On the contrary, we did not observe this effect in the expression of TGF-β superfamily members in P02 sera (Figure 3A).

Then, we analyzed the TGF-β bioactivity of P01’s and P02’s sera after the 1^st^ cell infusion, and compared them to that showed by the basal serum of each patient. Intriguingly, the TGF-β bioactivity of P01’s sera collected after the 1^st^ cell infusion showed a diminished trend when compared to that showed by P01’s basal serum, in spite of the enhanced expression of TGF-β superfamily members found in these sera (Figure 3B). Regarding P02, the sera collected after the 1^st^ cell infusion showed no TGF-β bioactivity (similar to the negative control), as occurred with the basal serum. These results were consistent with the absence of significant expression of TGF-β superfamily members in P02 sera before and after the 1^st^ cell infusion, (Figure 3B). Given that only the P01’s sera showed a modulation of TGF-β pathway after the 1^st^ cell infusion, we wondered if those sera collected after the successive MSCs administrations (second, third, fourth, fifth infusions) and in the follow-up visits (1 year and 2 years since the last cell infusion) in P01 exhibited also a reduction in the TGF-β bioactivity. Strikingly, we observed that the reduced TGF-β bioactivity of P01 sera was maintained with a similar trend during the consecutive MSCs infusions and follow-up visits (Figure 3C). These results suggest that, after the cell therapy, the circulating TGF-β inhibiting factors prevailed over those activating this pathway in P01.

## Discussion

The present study addresses, for the first time, the circulating TGF-β pathway in OI pediatric patients before and after receiving MSCs therapy. Our results should be interpreted in the context of certain limitations derived from the inherent nature of TERCELOI clinical trial. First, and the most important, the low number of pediatric patients included in this study, determined by the low prevalence of a rare disorder as OI and the restrictive inclusion criteria of TERCELOI such as the need of a HLA compatible sibling donor. Second, the fact that our patients were pediatric and third, the absence of a population of healthy pediatric controls. Thus, our findings need further confirmation in larger cohorts of OI patients, encompassing different OI severities and ages, along with age-matched healthy controls. Nevertheless, the present study provides new insights into the pathophysiology of OI, specifically concerning the circulating TGF-β pathway before and after MSCs therapy. Moreover, our observations shed light about the possibility of increased circulating levels of TGF-β superfamily members and TGF-β bioactivity correlating with OI severity.

Thus, to the best of our knowledge, this is the first time that an enhanced TGF-β expression and bioactivity has been reported in the serum of a severe OI pediatric patient when compared to a moderate OI one. These results are in line with previous findings in the skeleton of severe OI mice models and in osteoblasts isolated from OI patients (Gebken et al., 2000; Grafe et al., 2014; Tauer et al., 2018).

We speculate that the basal overactivation of circulating TGF-β pathway in the severe patient when compared to the moderate one could be due to the different phenotypic severities that these patients show, which in turn depends not only on the affected gene, but also on the type and the position of the mutation in the gene. In this line, mutations leading to a glycine substitution, such as that present in the severe patient, have been associated to most severe, and even perinatal lethal, phenotypes (Maioli et al., 2019). Thus, the enrichment of TGF-β superfamily members in the serum of the most severe patient could be reflecting the intense alteration in bone homeostasis that this patient exhibits. There are two evidences supporting this observation. First, the existence of increased circulating TGF-β1 activity and concentration in other connective tissue disorders such as heterotopic ossification, characterized by ectopic bone formation in extraskeletal tissues and Marfan syndrome, caused by mutations in fibrillin-1, an ECM protein (Matt et al., 2009; Franken et al., 2013; Wang et al., 2018). Second, as previously reported in TERCELOI, we also found striking molecular changes in basal serum from the most severe OI patient, which we also linked to the OI severity of this patient: an enhanced expression of circulating miRNAs known to be associated with osteoporotic bone fractures which was not observed in the moderate OI patient (Infante et al., 2021).

Interestingly, after the MSCs therapy we detected a general increase in the expression of several circulating TGF-β superfamily members only in the severe OI patient, but not in the moderate one. Surprisingly, TGF-β bioactivity of these sera was decreased when compared to that exerted by P01 basal serum. We speculate that this effect could be driven by the prevalence of ligands that function as inhibitors and compete with activators for the same TGF-β receptors, and the existence of more soluble TGF-β receptors (which can bind to ligands and therefore modulate the downstream TGF-β signaling) in the sera after cell therapy (Wang et al., 2017; Martinez-Hackert et al., 2021). Supporting our assumption, a previous study using the HEK-Blue-TGF-β^TM^ reporter cell line showed that not only the three TGF-β isoforms were able to activate the downstream TGF-β signaling, but also activin A, a member of the TGF-β superfamily binding to activing type I and II receptors, different from TGF-β receptors. Moreover, the concomitant addition of soluble chimeric TGF-β receptors inhibited this activation (Takahashi et al., 2020).

Importantly, our work shows that increased TGF-β bioactivity of basal serum from the severe OI patient can be modulated along the MSCs treatment, correlating with the beneficial effects exhibited by this patient (at clinical, molecular and cellular levels) after the cell therapy (Infante et al., 2021). Interestingly, the increased miRNA expression previously found in the basal serum of this patient, was also downregulated after the MSCs infusions.

The moderately affected patient exhibited low expression and bioactivity levels of circulating TGF-β, that were not modulated after the cell therapy. The specific mutation of this patient, an exon skipping of exon 35 in *COL1A2*, which leads to moderate OI, could be responsible for its lack of impact on the homeostasis of TGF-β pathway.

Assuming the limitations of the present study, our hypothesis linking OI disease severity with the activation of TGF-β pathway is in line with the increased skeleton TGF-β activation that mice models resembling severe OI, Col1a2^+/G61^°C, Crtap^−/-^, and Col1a1^Jrt/+^ exhibit (Grafe et al., 2014; Tauer et al., 2018). Interestingly, TGF-β inhibition was efficient in Col1a2^+/G61^°C, and Crtap^−/-^ mice, but not in Col1a1^Jrt/+^ ones, suggesting different effects of the antibody in different OI types or even different TGF-β activation levels in these OI murine models. Thus, OI severity should be a point of especial interest to take into account when addressing a TGF-β targeting approach.

On the other hand, it is worth mentioning that the inhibition of TGF-β has been also shown to be effective in mice models exhibiting skeletal pathologies and elevated TGF-β signaling, such as heterotopic ossification (HO), and osteoarthritis (Zhen et al., 2013; Wang et al., 2018). Moreover, and supporting our finding in the most severe patient, significantly elevated circulating levels of active TGF-β were found in HO patients (Wang et al., 2018). Denoting the interest in exploring the inhibition of TGF-β pathway in OI, a phase I clinical trial evaluating the safety and efficacy of fresolimumab, a human monoclonal antibody, that recognizes all TGF-β isoforms, is currently being assessed in adult patients (NCT03064074).

Overall, further studies involving representative cohorts of OI patients with different severities are needed to clarify the status of circulating TGF-β pathway activation in OI. Our study suggests that mainly severe OI patients would show increased circulating TGF-β signaling, and therefore could benefit from treatment approaches aiming to inhibit the excessive TGF-β activation.

The identification of those OI patients with excessive TGF-β would be crucial to select patients who may benefit from TGF-β targeting therapies.

## Conclusion

Osteogenesis Imperfecta (OI) is a rare collagen-related disorder with no current curative treatment. New therapeutic approaches have been tested with encouraging results, such as MSCs therapy. The inhibition of TGF-β signaling, effective in some severe murine OI models showing increased TGF-β activation in the skeleton, is also currently undergoing clinical evaluation for OI. This study examined for the first time the TGF-β pathway in the serum of two OI pediatric patients. These patients previously participated in TERCELOI clinical trial, and showed clinical improvements after receiving MSCs therapy. We found elevated basal TGF-β levels and bioactivity in the serum of the most severely affected patient, which were modulated after the MSCs therapy. The outcomes suggest that the specific mutation of the severe patient could be mediating the TGF-β overactivation. The current investigation provides a foundation for further exploring the circulating TGF-β pathway in a bigger cohort of OI patients encompassing different disease severities. The identification of OI patients with excessive TGF-β activation would be crucial to identify those patients who could benefit from TGF-β targeted therapies.

## Data Availability

The raw data supporting the conclusion of this article will be made available by the authors, without undue reservation.
